# Synthesis of the Key Intermediate of Coenzyme Q_10_

**DOI:** 10.3390/molecules16054097

**Published:** 2011-05-18

**Authors:** Fan-Song Mu, Meng Luo, Yu-Jie Fu, Xuan Zhang, Ping Yu, Yuan-Gang Zu

**Affiliations:** 1 Key Laboratory of Forest Plant Ecology, Ministry of Education, Northeast Forestry University, Harbin 150040, China; E-Mails: fansong2006@126.com (F.-S.M.); fuyujie1967@yahoo.com.cn (Y.-J.F.); 350330842@163.com (X.Z.); yup2010@163.com (P.Y.); 2 Engineering Research Center of Forestry Bio-preparation, Ministry of Education, Northeast Forestry University, Harbin 150040, China

**Keywords:** *p*-toluenesulfonyl chloride, isoprene, 2,3,4,5-tetramethoxytoluene, (2’*E*)-1-(3-methyl-4-*p*-toluenesulfonyl-2-butene)-6-methyl-2,3,4,5-tetramethoxybenzene

## Abstract

(2’*E*)-1-(3-methyl-4-*p*-toluenesulfonyl-2-butene)-6-methyl-2,3,4,5-tetramethoxybenzene (**4**) is the key intermediate in the synthesis of coenzyme Q_10_ *via* a coupling reaction with solanesyl bromide. In this paper, we report a simple and effective synthesis of compound **4**, starting with the readily available and inexpensive precursors *p*-toluenesulfonyl chloride (TsCl) and isoprene to obtain (2*E*)-1-*p*-toluenesulfonyl-2-methyl-4-hydroxy-2-butene (**3**) by addition, esterification and hydrolysis. Application of the Friedel-Crafts alkylation to compound **3**, followed by the addition of 2,3,4,5-tetramethoxytoluene (TeMT), assembled the two parts into compound **4**. The key parameters of each reaction were optimized at the same time, and the four total operations needed to produced compound **4** had a 27.9% overall yield under the optimized conditions. The structures of the compounds were characterized by ^1^H-NMR, IR and MS. This alternative process has the potential to be used for large-scale process.

## 1. Introduction

Coenzyme Q_10_ (CoQ_10_), consisting of a benzoquinone group and an isoprenoid chain, is a fat-soluble quinonoid compound that plays a pivotal role in several metabolic sequences. It exists in the mitochondria of every cell and participates in the electron transport process for respiration, thus producing energy for living organisms. It has also been used to treat heart-related diseases and has been used to enhance the immune system [1,2]. There is an increasing need for an efficient preparative method for this substance because of its remarkable physiological and clinical activity. A great deal of attention has been paid to CoQ_10_ since 1959. Over the years, researchers have put forward several semisynthetic strategies, most of which employ the side-chain extending method [3,4,5,6,7]. Using solanesol as a reagent for the synthesis of the all-*trans* isoprene side-chain of CoQ_10_ *via* coupling with quinone derivatives is a potentially valuable production method. Solanesol can readily be obtained by extraction from the leaves of tobacco or potato. However, its industrial application is still hampered by the lack of an efficient method for the preparation of quinone derivatives, which are the key intermediate of CoQ_10_. Therefore, a highly effective synthesis of quinone derivatives is important.

In particular, very promising key synthetic intermediates of CoQ_10_ have been developed. In 1979, Terao and co-workers demonstrated an efficient route to CoQ_10_ using a sulfone-functionalized prenylhydroquinone [8]. However, the stereoselective synthesis of the former component requires multistep procedures. Fujita *et al.* reported a new method that produces the phenyl sulfone side-chain *via* a Grignard reaction [9]. This multistep sequence forms the rather expensive key intermediate of CoQ_10_; however, the process still needs to be improved for industrial applications. A better procedure was proposed by Min *et al.* in 2003, who synthesized the key intermediate of CoQ_10_ *via* a Friedel-Crafts alkylation reaction, which greatly simplified the synthetic route [10]. However, this reaction required long reaction times and produced only low yields. New methods, such as those involving chloromethylated CoQ_0_ and ionic liquid, have been introduced into the synthesis of the key intermediate of CoQ_10_; however, these methods are still industrially inapplicable [11,12,13].

**Scheme 1:**
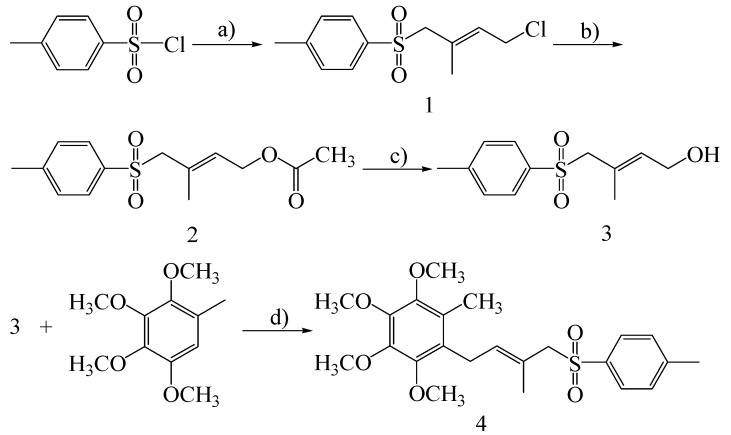
Synthesis of the key intermediate of CoQ_10_.

Here, we report a simple and effective synthesis of the key intermediate of CoQ_10_ (Scheme 1). The synthesis begins with the readily available and inexpensive precursors *p*-toluenesulfonyl chloride (TsCl) and isoprene to produce (2*E*)-1-*p*-toluenesulfonyl-2-methyl-4-hydroxy-2-butene (**3**) by addition, esterification and hydrolysis. The Friedel-Crafts alkylation was then applied to compound **3**, followed by the addition of 2,3,4,5-tetramethoxytoluene (TeMT), which assembles the two parts into (2’*E*)-1-(3-methyl-4-*p*-toluenesulfonyl-2-butene)-6-methyl-2,3,4,5-tetramethoxybenzene (**4**). Compound **4** is the key intermediate for the synthesis of CoQ_10_ *via* a coupling reaction with solanesyl bromide.

## 2. Results and Discussion

### 2.1. Synthesis of Compound ***1***

The reaction between isoprene and TsCl was catalyzed by copper (I) chloride-triethylamine hydrochloride at 90 °C for 6 h. Isoprene should be added with acetonitrile under a nitrogen atmosphere because it is highly volatile. The cocatalyst Et_3_N·HCl and the catalyst CuCl formed a CuCl alkyl amine salt, which caused the mixture to be a homogenous solution and inhibited the formation of dehydrochloride as a reaction product. After some exploratory experiments, we found suitable conditions (TsCl and CuCl) for a successful reaction (Table 1). The highest yield of compound **1** was achieved when the M_TsCl_:M_CuCl_ ratio was 1:0.15. However, the subsequent separation of compound **1** became more difficult with increasing amounts of CuCl. The optimal conditions for the formation of compound **1** were M_TsCl_:M_isoprene_:M_CuCl_:M_Et__3N·HCl_ = 1:1.8:0.15:0.15, heated at 90 °C for 6 h to produce compound **1** with a 75.9% yield.

### 2.2. Synthesis of Compound ***2***

Compound **1** and anhydrous sodium acetate were added to glacial acetic acid, and then the mixture was heated to the selected temperature. It should be noted that this reaction is quite sensitive to temperature. Five groups of experiments were carried out, and the results are summarized in Table 2. The maximum yield of compound **2** was achieved when the reaction temperature reached 120 °C. Dehydrochloride was formed when the temperature was increased to 125 °C and 130 °C. The optimized conditions found were M_compound **1**_:M_NaOAc_ = 1:1.8, heated at 120 °C for 5 h, which produced the desired compound **2** with a 90.7% yield. Furthermore, the product of this step can be used directly in the next reaction without any purification.

### 2.3. Synthesis of Compound ***3***

Compound **2** was dissolved in methanol and cooled down to 0 °C, then 20% sodium carbonate solution was added dropwise. Here, the addition time is an important factor in carrying out this step successfully. Sodium carbonate solution (20% sodium carbonate) must be added dropwise over 2 h; otherwise, the reaction will terminate if the addition time is too short. However, the yield of compound **3** did not change much when the addition time was within 2–3 h. Therefore, the optimal conditions were M_compound **2**_:M_Na2CO3_ = 1:1.6, 20% Na_2_CO_3_ added dropwise over 2 h at 0–5 °C, then reacted for 3 h to produce compound **3** with a 68.8% yield. 

### 2.4. Synthesis of Compound ***4***

Compound **4** was prepared by coupling 2,3,4,5-tetramethoxytoluene with compound **3** *via* a Friedel-Crafts reaction. The reaction of compound **3** with boron trifluoride etherate in 1,2-dichloroethane forms the corrersponding carbocation, then it attacks benzene to form a *δ*-complex. Finally, it loses a proton to form alkyl derivatives. Obviously, boron trifluoride etherate is an important factor for the synthesis of compound **4**. The color of the reactants sequentially changed from a light yellow, clear liquid to an orange and maroon color. From trial combinations of the precursor compound **3** and the catalyst boron trifluoride etherate (Table 4), the best results were obtained in the case of M_TeMT_:M_compound **3**_:M_BF3·OEt2_ = 1:1.2:0.3. With a slow increase of temperature to 80 °C and continuous stirring for 8 h, the yield of compound **4** rose to 58.8%. 

## 3. Experimental

### 3.1. General

All regents were analytical-grade chemicals and commercially available. The IR spectra were recorded with KBr pellets on a Nicolet Magna-560 spectrophotometer. The ^1^H-NMR spectra were recorded on a Bruker Avance-300 MHz instrument using CDCl_3_ as the deuterated solvent and TMS as the internal standard. Mass spectra were measured on an API-3000 LC/MS-MS (ESI).

### 3.2. (2E)-1-p-Toluenesulfonyl-2-methyl-4-chloride-2-butene *(**1**)*

TsCl (2 g, 10.499 mmol), CuCl (0.1567 g, 1.575 mmol) and Et_3_N·HCl (0.2165 g, 1.575 mmol) were mixed in four-neck round-bottom flask. A mixture of isoprene (1.89 mL, 18.9 mmol) and MeCN (4.2 mL) was added, and the reactant was stirred at 90 °C under a nitrogen atmosphere for 6 h. After completion, methanol (2 mL) was added to the mixture, which was cooled down to room temperature, then stirred and cooled to 0 °C, recrystallized and filtered to yield a deep yellow power. It was then dissolved in ethyl alcohol (1:2, m/v), cooled to 0 °C, filtered, dried under vacuum and recrystallized to produce compound **1** as white crystals with a yield of 75.9%. ^1^H-NMR (CDCl_3_) δ (ppm): 1.87 (s, 3H, CH_3_), 2.45 (s, 3H, CH_3_–Ph), 3.74 (s, 2H, CH_2_–SO_2_), 3.98 (d, 2H, *J* = 7.9 Hz, CH_2_–Cl), 5.32 (t, 1H, *J* = 7.9 Hz, =CH), 7.34 (d, 2H, *J* = 8.1 Hz, H–Ph), 7.73 (d, 2H, *J* = 8.1 Hz, H–Ph). IR (KBr), υ (cm^−1^): 2974, 2922, 1918, 1665, 1597, 1421, 1294, 1258, 1180, 1170, 1134, 1086, 890, 813, 752, 670, 629, 595, 543, 515, 483. MS (*m* / *z*): 281.3 [M + Na]^+^, 301.6 [M + MeOH]^+^.

### 3.3. (2E)-1-p-Toluenesulfonyl-2-methyl-4-acetoxy-2-butene *(**2**)*

Compound **1** (2 g, 7.737 mmol) and anhydrous sodium acetate (1.1424 g, 13.927 mmol) were added into a three-neck round-bottom flask, and glacial acetic acid (22.5 mL) was added. The mixture was then reacted at 120 °C for 5 h. After 5 h, the mixture was evaporated under reduced pressure to recycle 2/3 of the glacial acetic acid after the completion of the reaction. An appropriate amount of saturated sodium bicarbonate was added to the mixture to adjust the pH to 4. The mixture was then extracted with ethyl acetate (1:1, v/v) three times and evaporated under vacuum to obtain a faint yellow, oily liquid which was subjected to column chromatography over silica gel eluting with petroleum ether and ethyl acetate (5/1, v/v) to produce compound **2** in 90.7% yield as white crystals. ^1^H-NMR (CDCl_3_) δ (ppm): 1.84 (s, 3H, CH_3_), 2.02 (s, 3H, CH_3_–OAc), 2.46 (s, 3H, CH_3_–Ph), 3.74 (s, 2H, CH_2_–SO_2_), 4.99 (d, 2H, *J* = 6.8 Hz, CH_2_–OAc), 5.26 (t, 1H, *J* = 6.8 Hz, =CH), 7.34 (d, 2H, *J* = 8.1 Hz, H–Ph), 7.73 (d, 2H, *J* = 8.1 Hz, H–Ph). IR (KBr), υ (cm^−1^): 2975, 2923, 1732, 1597, 1372, 1307, 1298, 1270, 1235, 1131, 1170, 1131, 1086, 1026, 817, 747, 595, 539, 514, 486. MS (*m* / *z*): 305.1 [M + Na]^+^, 321.4 [M + K]^+^, 587.4 [2M + Na]^+^.

### 3.4. (2E)-1-p-Toluenesulfonyl-2-methyl-4-hydroxy-2-butene *(**3**)*

Compound **2** (2 g, 7.080 mmol) was dissolved in MeOH (12 mL) in a three-neck round-bottom flask, which was cooled down to 0 °C. Then, 20% Na_2_CO_3_ (6 mL, 11.327 mmol) was added dropwise over 2 h at 0 °C and stirred continuously for 3 h. The reaction was filtered and concentrated under reduced pressure after completion and extracted three times with ethyl acetate (1:1, v/v). The combined organic extraction was washed with water, dried over anhydrous sodium sulfate and evaporated under a reduced pressure to give an oily crude product. The crude was then dissolved in a suitable amount of ether, stirred and recrystallized at room temperature to isolate a white powder. The white powder was dissolved in petroleum ether by heating, then cooled to room temperature, recrystallized and filtered. This process was repeated until no other products could be detected by TLC. The product was dried under vacuum to produce compound **3** as a white solid with a yield of 68.8%. ^1^H-NMR (CDCl_3_) δ (ppm): 1.32 (s, 1H, OH), 1.82 (s, 3H, CH_3_), 2.45 (s, 3H, CH_3_–Ph), 3.73 (s, 2H, CH_2_–SO_2_), 4.11 (d, 2H, *J* = 6.3 Hz, CH_2_–OH), 5.33 (t, 1H, *J* = 6.3 Hz, =CH), 7.35 (d, 2H, *J* = 8.1 Hz, H–Ph), 7.74 (d, 2H, *J* = 8.1 Hz, H–Ph). IR (KBr), υ (cm^−1^): 3365, 2975, 2923, 1597, 1311, 1142, 1087, 1009, 817, 733, 677, 587, 537. MS (*m* / *z*): 263.4 [M + Na]^+^, 279.2 [M + K]^+^, 503.2 [2M + Na]^+^.

### 3.5. (2’E)-1-(3-Methyl-4-p-toluenesulfonyl-2-butene)-6-methyl-2,3,4,5-tetramethoxymethylbenzene *(**4**)*

2,3,4,5-Tetramethoxytoluene (TeMT, 1 g, 4.717 mmol) and compound **3** (1.3585 g, 5.660 mmol) were dissolved in 1,2-dichloroethane (10 mL). BF_3_·OEt_2_ (0.18 mL, 1.415 mmol) was then added, and the temperature was slowly increased to 80 °C. The reaction was allowed to proceed for 8 h. NH_4_Cl solution was added to the mixture after the completion of the reaction, and the reaction product was left standing to separate the organic phase and the water phase. The organic layers were washed with 5% HCl, brine and distilled water in sequence, then dried over anhydrous sodium sulfate, filtered and concentrated under a reduced pressure to reveal a yellow, oily product. The oily residue was purified by silica gel column chromatography using petroleum ether and ethyl acetate (10/1, v/v) as the eluent to afford compound **4** as a white solid in 58.8% yield. ^1^H-NMR (CDCl_3_) δ (ppm): 1.94 (s, 2H, CH_2_–SO_2_), 1.99 (s, 3H, CH_3_), 2.40 (s, 3H, CH_3_–Ph), 3.25 (d, 2H, *J* = 6.3 Hz, CH_2_–Ph), 3.69–3.91 (m, 15H, OCH_3_ × 4–Ph, CH_3_–Ph), 4.92 (t, 1H, *J* = 6.3 Hz, =CH), 7.23 (d, 2H, *J* = 8.1 Hz, H–Ph), 7.66 (d, 2H, *J* = 8.1 Hz, H–Ph). IR (KBr), υ (cm^−1^): 2976, 2933, 1597, 1470, 1406, 1352, 1312, 1162, 1132, 1109, 1087, 1069, 1040, 965, 892, 742, 623, 581, 532, 510. MS (*m* / *z*): 435.3 [M + H]^+^, 457.1 [M + Na]^+^, 452.4 [M + NH_4_]^+^, 891.5 [2M + Na]^+^.

## 4. Conclusions

In the present study, we have developed a simple and effective method for the synthesis of compound **4**, the key intermediate of CoQ_10_. The key parameters of each reaction were also optimized and the overall yield of compound **4** was 27.9% under the optimized conditions (calculated based on TsCl). The success of this approach is presumably due to the stabilization provided by a *p*-phenylsulfonyl group in compound **3** in the presence of Lewis acids. The *E*-configuration of the C=C bond was mostly retained in compound **4** after the Friedel-Crafts reaction. Furthermore, this method produced the key intermediate of CoQ_10_ from the relatively inexpensive precursor isoprenol, which is more economical than expanding the expensive natural nonaprenyl compound, solanesol, by an isoprene unit before coupling it to the CoQ_10_ precursor. The present process has the potential to be used for large-scale process.

## Figures and Tables

*Reagents and conditions*: (a): isoprene, copper (I) chloride (CuCl), triethylamine hydrochloride (Et_3_N·HCl), 90 °C, 75.9%; (b): NaOAc, glacial acetic acid, 120 °C, 90.7%; (c): MeOH, 20% Na_2_CO_3_, 0 °C, 68.8%; (d): 2,3,4,5-tetramethoxytoluene (TeMT), 1,2-dichloroethane, boron trifluoride etherate (BF_3_·OEt_2_), 80 °C, 58.8%.

**Table 1:** Effect of CuCl on the yield of compound **1**.

Number	1	2	3	4	5	6
CuCl / g	0.052	0.105	0.157	0.209	0.313	0.522
Yield / %	-	58.6	75.9	75.0	70.4	68.8

**Table 2:** Effect of reaction temperature on the yield of compound **2**.

Number	1	2	3	4	5
Temperature / °C	110	115	120	125	130
Yield / %	53.3	72.0	90.7	80.4	62.6

**Table 3:** The effect of the addition time of 20% sodium carbonate solution on the yield of compound **3**.

Number	1	2	3	4	5	6
Na_2_CO_3_ adding time / h	0.5	1	1.5	2	2.5	3
Yield / %	20.1	43.4	52.8	68.8	68.7	68.7

**Table 4:** Effect of boron trifluoride etherate on the yield of compound **4**.

Number	1	2	3	4	5
BF_3_·OEt_2_ / mL	0.06	0.12	0.18	0.24	0.3
Yield / %	-	32.2	58.8	44.8	29.4

## References

[1] Anderson S.S., Lyle I.G., Paterson R. (1976). Electron transfer across membranes using vitamin K_1_ and coenzyme Q_10_ as carrier molecules. Nature.

[2] Littarru G.P., Tiano L. (2010). Clinical aspects of coenzyme Q_10_: An update. Nutrition.

[3] Rüegg R., Gloor U., Goel R.N., Ryser G., Wiss O., Isler O. (1959). Synthesis of bichinon(45) and ubichinon(50). Helv. Chim. Acta.

[4] Yoshizawa T., Toyofuka H., Tachibana K., Kuroda T. (1982). Regioselective polyprenyl rearrangement of polyprenyl 2,3,4,5-tetrasubstituted phenyl ethers promoted by boron trifluoride. Chem. Lett..

[5] Van Liemt W.B.S., Steggerda W.F., Esmeijer R., Lugtenburg J. (1994). Synthesis and spectroscopic characterization of ^13^C-labelled ubiquinone-0 and ubiquinone-10. Recl. Trav. Chim. Pays-Bas..

[6] Lipshutz B.H., Mollard P., Pfeiffer S.S., Chrisman W.A. (2002). Short, highly efficient synthesis of coenzyme Q_10_. J. Am. Chem. Soc..

[7] Negishi E., Liou S.Y., Xu C., Huo S. (2002). A novel, highly selective, and general methodology for the synthesis of 1,5-diene-containing oligoisoprenoids of all possible geometrical combinations exemplified by an iterative and convergent synthesis of coenzyme Q_10_. Org. Lett..

[8] Terao S., Kato K., Shiraishi M., Morimoto H. (1979). Synthesis of ubiquinones. 2. An efficient preparation of ubiquinone-10. J. Org. Chem..

[9] Fujita Y., Ishiguro M., Onishi T., Nishida T. (1982). A new efficient and stereoselective synthesis of ubiquinone-10. Bull. Chem. Soc. Jpn..

[10] Min J.H., Lee J.S., Yang J.D., Koo S. (2003). The Friedel-Crafts alkylation of a prenyl group stabilized by a sulfone moiety: Expeditious synthesis of ubiquinones and menaquinones. J. Org. Chem..

[11] Lipshutz B.H., Lower A., Berl V., Schein K., Wetterich F. (2005). An improved synthesis of the “Miracle Nutrient”, coenzyme Q_10_. Org. Lett..

[12] Ravada S.R., Emani L.R., Garaga M.R., Meka B., Golakoti T. (2009). Synthesis of coenzyme Q_10_. Am. J. Infect. Dis..

[13] Chen Y., Zu Y.G., Fu Y.J., Zhang X., Yu P., Sun G.Y., Efferth T. (2010). Efficient lewis acid ionic liquid-catalyzed synthesis of the key intermediate of coenzyme Q_10_ under microwave irradiation. Molecules.

